# Determinants of women’s empowerment in Pakistan: evidence from Demographic and Health Surveys, 2012–13 and 2017–18

**DOI:** 10.1186/s12889-021-11376-6

**Published:** 2021-07-06

**Authors:** Safdar Abbas, Noman Isaac, Munir Zia, Rubeena Zakar, Florian Fischer

**Affiliations:** 1 Department of Sociology, Institute of Social & Cultural Studies, University of the Punjab, Lahore, Pakistan; 2 Department of Public Health, Institute of Social & Cultural Studies, University of the Punjab, Lahore, Pakistan; 3 Institute of Public Health, Charité – Universitätsmedizin Berlin, Berlin, Germany; 4 Institute of Gerontological Health Services and Nursing Research, Ravensburg-Weingarten University of Applied Sciences, Weingarten, Germany

**Keywords:** Decision-making, Autonomy, Ownership, Reproductive age, Well-being

## Abstract

**Background:**

Women’s empowerment has always remained a contested issue in the complex socio-demographic and cultural milieu of Pakistani society. Women are ranked lower than men on all vital human development indicators. Therefore, studying various determinants of women’s empowerment is urgently needed in the Pakistani context.

**Methods:**

The study empirically operationalized the concept of women’s empowerment and investigated its determinants through representative secondary data taken from the Pakistan Demographic and Health Surveys among women at reproductive age (15–49 years) in 2012–13 (*n* = 13,558) and 2017–18 (*n* = 15,068). The study used simple binary logistic and multivariable regression analyses.

**Results:**

The results of the binary logistic regression highlighted that almost all of the selected demographic, economic, social, and access to information variables were significantly associated with women’s empowerment (*p* < 0.05) in both PDHS datasets. In the multivariable regression analysis, the adjusted odds ratios highlighted that reproductive-age women in higher age groups having children, with a higher level of education and wealth index, involved in skilled work, who were the head of household, and had access to information were reported to be more empowered.

Results of the multivariable regression analysis conducted separately for two empowerment indicators (decision-making and ownership) corroborated the findings of the one indicator of women empowerment, except where ownership did not appear to be significantly associated with number of children and sex of household head in both data sets (2012–13 and 2017–18).

**Conclusions:**

A number of social, economic, demographic, familial, and information-exposure factors determine women’s empowerment. The study proposes some evidence-based policy options to improve the status of women in Pakistan.

## Background

Women’s empowerment per se involves the creation of an environment within which women can make strategic life choices and decisions in a given context [1]. The concept is so broad that measuring it has always been problematic. Following from this conundrum, various studies have developed different conceptualisation schemes and indicators to measure the complex idea [2]. For instance, women’s empowerment depends upon cultural values, the social position, and life opportunities of a woman [3]. Women’s empowerment can take place on three dimensions, which are at the micro-level (individual), meso-level (beliefs and actions in relation to relevant others), and macro-level (outcomes in the broader, societal context) [4]. Furthermore, women’s empowerment could be characterized in four major domains: socio-cultural, economic, education, and health [5]. While differences exist in measuring the concept of empowerment, similarities can be found in the available literature. In this regard, the main themes frequently used to conceptualize women’s empowerment are household decision-making, economic decision-making, control over resources, and physical mobility [6–9].

From this point of departure, the present study attempts to identify and understand various determinants of women’s empowerment in Pakistani society with the help of representative data from Demographic and Health Surveys. Investigating women’s empowerment in Pakistan is important, because of the male dominance and gender gaps which are hindering the progress of women to take an active part in development in Pakistani society [10]. Furthermore, empowerment is a strong determinant for healthcare decision-making as well as of physical and mental health in females [11].

Because women’s empowerment is an idea that acknowledges a woman’s control over her own life and personal decisions, it has a strong grounding in human rights propositions [1]. Moreover, women constitute almost half the world’s population; hence, women’s empowerment is the key factor in achieving the highest levels of desirable development [12].

Despite the widespread acclamation of women’s empowerment and the major role of women in the development process, their status is not equal to that of men across most countries of the world [13]. In many parts of the world, women are in a disadvantaged position, and hence most of the time ranked below their male counterparts in the social hierarchy [14]. This disadvantaged position can well be understood through the glaring differences between men and women with respect to many human-rights, cultural, economic, and social indicators. For instance, globally, women spend two to ten times more hours than men on unpaid care work [15]. Similarly, of all the illiterate and poor people across the world, women constitute 65 and 70% respectively [16]. It is reported that only 1% of the world’s total assets are held in women’s names [17]. Moreover, data also indicates that 70% of the 1.3 billion people living in extreme poverty are women or girls [18]. Owing to these conditions, women enjoy substantially lower status than men [15].

Although gender-based discrimination is a global issue, Pakistan needs special attention in terms of women’s empowerment [19]. Pakistani society, in both its normative and existential order, is hierarchical in nature and exhibits unequal power relations between men and women, whereby women are placed under men [20]. The existence of significant gender disparities makes it a non-egalitarian society where gender equality and women’s emancipation appear a faraway goal [21]. In this context, the low level of women’s empowerment is a factual issue in Pakistan as the country is ranked almost at the bottom of the Gender Gap Index – 151st of 153 studied countries [22]. Similarly, in 2019, the Human Development Index value for females was lower than for males (0.464 vs. 0.622) in the country [23].

The gender disparity highlighted by these measures can be clearly observed through the evidence at hand. For instance, Pakistan has a very low rate of female labour-force participation compared to their male counterparts (25% vs. 82%) [24]. In addition, adult women had less secondary-school education than males (26.7% vs. 47.3%) [23]. Concomitantly, low educational opportunities and poor educational achievement lead to low empowerment among women, particularly those who live in remote areas of the country [25, 26]. The situation is further exacerbated when female parliamentarians in Pakistan appear to be bound by patriarchal beliefs and practices when they could realize empowerment. In such circumstances, the notion of empowerment in Pakistan appears to be only theoretical without any sense of practical embodiment [27].

**Fig. 1:**
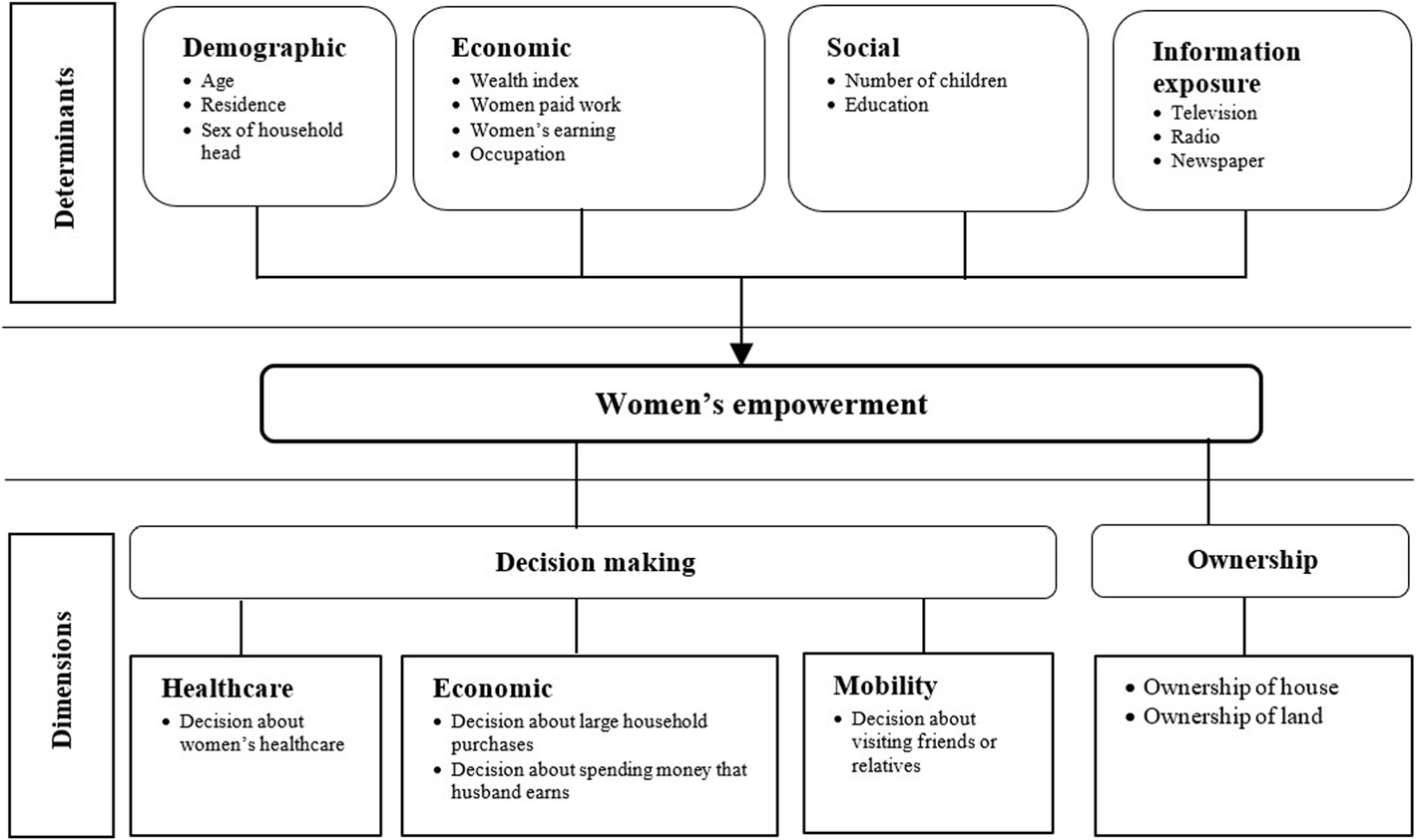
Conceptualization of determinants and dimensions of women’s empowerment

Against this backdrop of a persistently bleak situation for women’s empowerment in the country, the government of Pakistan has launched some targeted actions, such as the National Policy of Development and Empowerment in 2002, which aimed to improve the economic, social, and political empowerment of women. Additionally, the number of seats reserved for women in both the Senate and the National and Provincial Assemblies has also been increased. Nevertheless, women in Pakistan are still subjected to unequal power relations, and are less authorized to make decisions about their own lives [28]. The country stands among the lowest in the world in terms of women’s empowerment, even though almost half its population is made up of women, and empowering them could improve the overall well-being of society. There is a paucity of literature empirically conceptualising women’s empowerment and its determinants in Pakistan. For that reason, we have adapted the framework developed by Mahmud et al. [8], which conceptualizes women’s empowerment as a dynamic and multi-dimensional process. By the same token, the framework of the present study encompasses four major determinants: demographic, economic, social, and information-exposure factors. Likewise, it denotes two major dimensions of women empowerment, which are decision-making and ownership. Decision-making involves decisions about healthcare, economic affairs, and mobility issues. Ownership includes the ownership of house and land. Conceptualizing the determinants and dimensions of women’s empowerment with empirical and representative data is the unique aspect of the study, which adds to the body of knowledge. The theoretical framework used to explain the link between the determinants and dimensions of women’s empowerment is given in Fig. 1. The results of the present study help to present policy implications for enhancing women’s status in Pakistan.

## Methods

This study is based on secondary data from the two nationally representative Pakistan Demographic and Health Surveys (PDHSs) 2012–13 and 2017–18 [29]. These are the third and fourth such surveys conducted as part of the MEASURE DHS International Series, whose sample was selected with the help of the Pakistan Bureau of Statistics. The present study used the secondary data of PDHS 2012–13 and 2017–18, drawn by two-stage stratified sample design, consisting of 13,558 and 15,068 currently ever-married women aged 15–49 years, respectively. Both PDHSs deployed a cross-sectional study design with the primary objective to provide up-dated estimates on basic demographic, health, and domestic violence indicators. The present study used data from the woman’s questionnaire.

### Variables: definitions and construction

In this study, we drew variables from the PDHS data sets of 2012–13 and 2017–18 available in SPSS format. In this regard, women’s empowerment was assessed using two variables, on decision-making and ownership. To measure decision-making, we computed four variables, concerning decision-making about: “spending money husband earns”, “major household purchases”, “women’s healthcare”, and “visiting family or relatives”. Each of these four decision-making variables had six response categories; namely: “respondent alone” coded as 1, “respondent and husband/partner” coded as 2, “respondent and other person” coded as 3, “husband/partner alone” coded as 4, “someone else” coded as 5, and “other/family elders” coded as 6. For each of the four decision-making variables, data was categorized as women “not involved in decision-making”, recoded as “0”, when the woman was not involved in decision-making at all, and “involved in decision-making”, recoded as “1”, when the woman was involved in any of the four variables of decision-making. Subsequently, all the four recoded variables were computed into one variable of “decision-making” with dichotomous categories of “No” coded “0” and “Yes” coded “1” for any kind of involvement in decision-making.

Women’s ownership of property was computed using two variables: a woman “owns a house alone or jointly” and/or “owns land alone or jointly”. We computed these variables into one variable and recoded “0” if a woman did not own a house/land, alone or jointly, and “1” if she did own a house/land, alone or jointly. The two variables “decision-making” and “ownership” were computed into one variable, i.e. “women’s empowerment”, and recoded into two response categories: “not empowered” coded as “0” if the woman was not at all involved in household decision-making and did not possess a house/land, and “empowered” as “1” if the woman was involved in decision-making and/or owned a house/land. This variable was used as the dependent variable in the regression analysis with the various independent variables concerning demographic, economic, and social status, along with access to information. A separate multivariable regression analysis was also conducted to see the associations between independent variables and both indicators for women’s empowerment, which are 1) decision-making and 2) ownership.

The present study used independent variables related to socio-demographic characteristics (age, area of residence, and sex of household head), economic (wealth index, women’s paid work, women’s earnings, and women’s occupation) as well as social factors (number of children, women’s education, and husband’s education) and access to information (frequency of watching TV, frequency of listening to radio, and frequency of reading newspapers).

The wealth index is a composite measure of a household’s cumulative living standard. It is calculated using easy-to-collect data and allows to distribute into wealth quintiles. The wealth index was measured using monthly income and household possessions, which are total value of household assets, availability of household items such as a car or refrigerator, value of dwelling, and other civic facilities, including access to safe drinking water, sanitation facilities, and dwelling characteristics. Employment status was assessed during the previous 12 months and afterwards dichotomized into “paid” and “unpaid” work categories.

We created a new variable: “access to information”, by computing three categorical variables: “frequency of watching TV”, “frequency of listening to radio”, and “frequency of reading newspapers”. Responses were categorized as “0” if women had “no access” to any source, and “1” if women had access to at least one source of information either daily, weekly, or occasionally. Two separate copies of SPSS files (2012–13 and 2017–18) were generated consisting of all recoded and computed variables to run requisite analyses.

### Data analysis

The data were analysed by using SPSS 21. Descriptive statistics were performed. We ran a simple binary logistic regression analysis to examine the association between women’s empowerment and each of the independent variables in turn. After running the simple binary logistic regression for calculating odds ratios (OR), we applied multivariable logistic regression to predict the dependent variables through independent variables, while adjusting for region, income, and employment. Adjusted odds ratios (AOR) and their 95% confidence intervals (CI) have been calculated. We tested for multicollinearity.

## Results

### Sample characteristics

The results from the two datasets, taken from PDHS 2012–13 and PDHS 2017–18, corroborated each other. The mean age of the respondents was almost the same in 2012–13 and 2017–18 (32.7 vs. 32.1 years). Similarly, the majority of ever-married women had children. In nearly all households, males were indicated as the household head (91.5% in 2012–13 and 89.0% in 2017–18). The results indicated that there was a slight improvement in education, with 56.2% being uneducated in 2012–13, reducing to 50.6% in 2017–18. The data revealed that more than three-quarters of women during both 2012–13 and 2017–18 had not done any paid work during the previous 12 months (78.0% vs. 84.6%). Among the total responses about earnings (2243 in 2012–13 and 1866 in 2017–18), only 18.1 and 17.0% of working women, respectively, were earning more than their husbands. Just over two-thirds (67.9%) of women had no access to sources of information (such as TV, radio, or newspapers) in 2012–13, and this figure had increased to 80.6% in 2017–18 (Table 1).

**Table 1:** *Sample characteristics (n = 13,558 in PDHS 2012–13 and n = 15,068 in PDHS 2017–18)*

	PDHS 2012–13		PDHS 2017–18	
	n	%	*n*	%
**Age in years**^**a**^				
15–19	567	4.2	728	4.8
20–24	2048	15.1	2220	14.7
25–29	2723	20.1	3146	20.9
30–34	2438	18.0	2853	18.9
35–39	2300	17.0	2738	18.2
40–44	1808	13.3	1821	12.1
45–49	1674	12.3	1562	10.4
**Type of place of residence**				
Urban	6351	46.8	7254	48.1
Rural	7207	53.2	7814	51.9
**Sex of household head**				
Male	12,409	91.5	13,412	89.0
Female	1149	8.5	1656	11.0
**Marital status**^**b**^				
Living with partner	13,010	96.0	14,502	96.2
Without partner	548	4.0	566	3.8
**Wealth index**				
Poorest	2486	18.3	2886	19.2
Poorer	2586	19.1	3240	21.5
Middle	2589	19.1	2966	19.7
Richer	2657	19.6	2878	19.1
Richest	3240	23.9	3098	20.6
**Employment**				
No paid work	10,567	78.0	12,745	84.6
Paid work in last 12 months	2975	22.0	2320	15.4
**Earning**				
Earns less than husband	1838	81.9	1548	83.0
Earns more than husband	405	18.1	318	17.0
**Occupation of respondent**				
Unemployed	10,591	78.1	12,748	84.6
Unskilled	1491	11.0	999	6.6
Skilled	1085	8.0	791	5.2
Managerial	390	2.9	522	3.5
**Number of children**				
No children	1695	12.5	2048	13.6
1–3 children	5957	43.9	7096	47.1
4–6 children	4412	32.5	4682	31.1
7–9 children	1321	9.7	1088	7.2
≥ 10 children	173	1.3	154	1.0
**Respondent’s education**				
No education	7625	56.2	7627	50.6
Primary	1831	13.5	2103	14.0
Secondary	2415	17.8	3132	20.8
Higher	1687	12.4	2206	14.6
**Husband’s education**				
No education	4215	31.1	4007	27.6
Primary	1819	13.4	1922	13.3
Secondary	4301	31.8	5094	35.1
Higher	3176	23.0	3474	24.0
**Access to information**				
No	9169	67.9	12,148	80.6
Yes	4344	32.1	2918	19.4
**Region**				
Punjab	3800	28.0	3400	22.6
Sindh	2941	21.7	2739	18.2
KPK	2695	19.9	2378	15.8
Balochistan	1953	14.4	1724	11.4
Gilgit-Baltistan	1216	9.0	984	6.5
Islamabad (ICT)	953	7.0	1111	7.4
Azad Jammu Kashmir	–		1720	11.4
Federally Administered Tribal Areas	–		1012	6.7

^a^ Standard deviation + 8.54; Mean 32.69 for 2012–13 / Standard deviation + 8.43; Mean 32.11 for 2017–18

^b^ including separated, divorced and widowed women

### Decision-making, ownership, and empowerment

Decision-making about healthcare showed mixed results, with almost half of the women (48.1% in 2012–13 and 48.2% in 2017–18) being involved in this domain of decision-making. In both 2012–13 and 2017–18, around half of the women (47.1% vs. 46.4%) were involved in decision-making about visiting family or relatives. Likewise, in 2012–13 and 2017–18, more than half of women (56.9% vs. 58.5%) were not involved in decision-making about large household purchases. Comparably, not being involved in decision-making regarding spending the money earned by their husband was a little higher in 2012–13 than in 2017–18 (59.7% vs. 50.2%). The vast majority of women did not own a house or land in either 2012–13 or 2017–18 (82.3% vs. 82.6%). Thus, the data indicates that more than half of the women in 2012–13 and 2017–18 were reported as not being empowered (58.4% vs. 53.2%) (Table 2).

**Table 2:** *Decision-making, ownership, and empowerment at household (n = 13,558 in PDHS 2012*–*13 and n = 15,068 in PDHS 2017*–*18)*

	PDHS 2012–13		PDHS 2017–18	
	n	%	n	%
**Decision- making about healthcare**				
Not involved in decision-making	6746	51.9	7507	51.8
Involved in decision-making	6243	48.1	6993	48.2
**Decision-making about large household purchases**				
Not involved in decision-making	7393	56.9	8488	58.5
Involved in decision-making	5599	43.1	6012	41.5
**Decision-making about the money husband earns**				
Not involved in decision-making	7716	59.7	6203	50.2
Involved in decision-making	5201	40.3	6161	49.8
**Decision-making about visits to relatives**				
Not involved in decision-making	6878	52.9	7767	53.6
Involved in decision-making	6114	47.1	6733	46.4
**House/land ownership**				
No ownership	11,142	82.3	12,440	82.6
Ownership	2392	17.7	2622	17.4
**Empowerment**				
No	7535	58.4	6578	53.2
Yes	5367	41.6	5781	46.8

### Simple binary logistic regression

We used simple binary logistic regression to find the prediction for each of the independent variables on the dependent variable in both datasets. It was found that the likelihood of empowerment increased with an increase in the woman’s age. Similarly, in relation to the wealth index, the likelihood of empowerment was highest for the richest women. Likewise, the data also highlighted that women earning more than their husbands were more likely to be empowered than those earning less (OR = 2.00, 95% CI: 1.59–2.52 in 2012–13; OR = 1.64, 95% CI: 0.66–4.04 in 2017–18). The data indicated that women with higher education were more empowered (OR = 2.20, 95% CI: 1.97–2.45 in 2012–13; OR = 1.69, 95% CI: 1.44–1.99 in 2017–18) than women with no or less education. The simple binary logistic regression also showed that almost all of the predictor variables were significantly associated (*p* < 0.05) with women’s empowerment (Table 3).

**Table 3:** *Simple binary logistic regression analysis of factors associated with women empowerment (n = 13,558 in PDHS 2012*–*13 and n = 15,068 in PDHS 2017*–*18)*

	PDHS 2012–13			PDHS 2017–18		
Variables	OR	95% CI		OR	95% CI	
		Lower	Upper		Lower	Upper
**Age in years**						
15–19 (reference)						
20–24	2.11***	1.61	2.76	1.20	0.93	1.54
25–29	3.39***	2.60	4.40	1.64***	1.28	2.09
30–34	5.09***	3.92	6.62	2.17***	1.69	2.79
35–39	6.97***	5.36	9.06	2.45***	1.90	3.16
40–44	9.70***	7.43	12.68	3.02***	2.31	3.93
–49	9.23***	7.05	12.09	3.85***	2.93	5.06
**Type of place of residence**						
Rural (reference)						
Urban	1.65***	1.54	1.77	0.93	0.85	1.02
**Sex of household head**						
Male (reference)						
Female	2.02***	1.76	2.32	2.42***	2.11	2.79
**Wealth index**						
Poorest (reference)						
Poorer	1.68***	1.49	1.90	1.33***	1.17	1.52
Middle	1.88***	1.66	2.12	1.35***	1.17	1.56
Richer	2.20***	1.96	2.48	1.35***	1.15	1.59
Richest	2.82***	2.51	3.16	1.35*	1.13	1.62
**Employment**						
No paid work (reference)						
Paid work in last 12 months	1.70***	1.56	1.85	1.46***	1.33	1.66
**Earning**						
Earns less than husband (ref)						
Earns more than husband	2.00***	1.59	2.52	1.64**	0.66	4.04
**Occupation of respondent**						
Unemployed (reference)						
Unskilled	1.39***	1.24	1.56	1.18*	0.99	1.40
Skilled	1.70***	1.49	1.94	1.56***	1.33	1.84
Managerial	3.92***	3.11	4.94	1.96***	1.54	2.49
**Number of children**						
No children (reference)						
1–3 children	2.20***	1.94	2.50	1.29***	1.12	1.47
4–6 children	3.46***	3.03	3.95	1.35***	1.16	1.57
7–9 children	2.85***	2.42	3.35	1.05	0.85	1.28
≥ 10 children	2.06***	1.46	2.91	0.93	0.62	1.38
**Respondent’s education**						
No education (reference)						
Primary	1.33***	1.20	1.48	1.25***	1.10	1.42
Secondary	1.46***	1.33	1.61	1.30***	1.15	1.47
Higher	2.20***	1.97	2.45	1.69***	1.44	1.99
**Husband’s education**						
No education (reference)						
Primary	1.05	0.94	1.18	0.87	0.76	0.99
Secondary	1.09	1.00	1.20	0.98	0.88	1.10
Higher	1.55***	1.41	1.71	1.15*	1.00	1.31
**Access to information**						
No (reference)						
Yes	1.65***	1.53	1.78	1.16**	1.05	1.30
**Region**						
Punjab (reference)						
Sindh	0.66***	0.60	0.73	1.35***	1.20	1.53
KPK	0.40***	0.36	0.45	0.38***	0.33	0.43
Balochistan	0.32***	0.29	0.36	0.37***	0.32	0.43
Gilgit-Baltistan	0.54***	0.47	0.62	0.61***	0.51	0.73
Islamabad (ICT)	1.21***	1.04	1.40	1.13*	0.97	1.33
Azad Jammu Kashmir	–			0.98	0.85	1.12
Federally Administered Tribal Areas	–			0.16	0.12	0.21

OR = Odds ratio, CI = Confidence interval (*p < 0.05; **p < 0.01; ***p < 0.001)

### Multivariable logistic regression analysis

The results of multivariable logistic regression model indicated that, after adjustment, almost all of the predictor variables were significantly associated with “decision-making” and most of predictor variables with “ownership”. Data indicated that women in the higher age group (45–49) were more involved in decision-making (AOR = 4.51, 95% CI: 2.31–9.26 in 2012–13; AOR = 3.72, 95% CI: 2.01–6.91 in 2017–18) and had ownership (AOR = 1.20, 95% CI: 0.94–1.52 in 2012–13; AOR = 3.72, 95% CI: 2.01–6.91 in 2017–18) compared to their counterparts. Females as household heads showed a significant association with decision-making (AOR = 2.09, 95% CI: 1.79–2.44 in 2012–13; AOR = 2.52, 95% CI: 2.21–2.87 in 2017–18) but it did not appear to be significantly associated with ownership in both data sets. Likewise, the number of children had a significant association with decision-making but not with ownership. Data also revealed that higher education of women was significantly associated with decision-making (AOR = 2.01, 95% CI: 1.73–2.34 in 2012–13; AOR = 2.23, 95% CI: 1.91–2.61 in 2017–18) and ownership (AOR = 1.51, 95% CI: 1.26–1.80 in 2012–13; AOR = 2.08, 95% CI: 1.48–2.91 in 2017–18). Access to information also appeared to be associated with decision-making and ownership (Table 4).

**Table 4:** *Multivariable logistic regression of factors associated with decision-making and ownership (n = 13,558 in PDHS 2012*–*13 and n = 15,068 in PDHS 2017*–*18)*

	PDHS 2012–13						PDHS 2017–18					
	Decision-making			Ownership			Decision-making			Ownership		
	AOR	95% CI		AOR	95% CI		AOR	95% CI		AOR	95% CI	
		Lower	Upper		Lower	Upper		Lower	Upper		Lower	Upper
**Age in years**												
15–19 (reference)												
20–24	1.03*	0.59	2.15	0.740	0.58	0.94	1.40*	1.09	1.79	1.07	0.58	1.96
25–29	2.42***	2.2	2.66	0.74	0.59	0.94	1.94***	1.52	2.47	1.49	0.83	2.67
30–34	3.00***	1.54	5.86	0.85	0.67	1.08	2.71***	2.12	3.47	1.32	0.72	2.42
35–39	4.26***	2.15	8.43	0.98*	0.78	1.24	2.97***	2.32	3.82	2.48*	1.37	4.49
40–44	3.90***	3.38	4.55	1.09*	0.86	1.38	3.67***	2.83	4.76	2.14*	1.15	4.21
45–49	4.51***	2.31	9.26	1.2**	0.94	1.52	4.82***	3.7	6.29	3.72***	2.01	6.91
**Type of place of residence**												
Rural (reference)												
Urban	0.69***	0.63	0.77	1.35***	1.19	1.52	0.92***	0.91	0.94	0.99	0.95	1.03
**Sex of household head**												
Male (reference)												
Female	2.09***	1.79	2.44	1.07	0.89	1.28	2.52***	2.21	2.87	1.18	0.89	1.56
**Wealth index**												
Poorest (reference)												
Poorer	1.39***	1.23	1.59	1.40***	1.19	1.66	1.24***	1.10	1.41	1.09	0.74	1.61
Middle	1.47***	1.29	1.69	1.37**	1.14	1.64	1.41***	1.23	1.61	1.60*	1.09	2.36
Richer	1.30***	1.13	1.52	1.57***	1.28	1.91	1.57***	1.36	1.82	1.73*	1.15	2.59
Richest	1.16	0.98	1.38	2.70***	2.16	3.37	1.73***	1.48	2.03	2.19***	1.43	3.34
**Occupation of respondent**												
Unemployed (reference)												
Unskilled	1.27*	1.43	2.04	1.34***	1.09	2.01	1.94***	1.66	2.28	1.03	0.67	1.59
Skilled	1.51***	1.26	2.01	1.13***	1.01	1.29	1.44***	1.22	1.70	1.19	0.8	1.78
Managerial	2.01***	1.33	2.21	1.91***	1.45	2.32	2.08***	1.67	2.59	1.63**	1.19	2.27
**Number of children**												
No children (reference)												
1–3 children	2.15***	1.89	2.45	1.13	0.95	1.33	1.22*	1.07	1.39	0.84	0.64	1.11
4–6 children	2.99***	2.59	3.47	1.1	0.91	1.33	1.09*	0.95	1.27	0.74	0.54	1.03
7–9 children	2.29***	1.91	2.76	1.09	0.86	1.38	0.72**	0.59	0.88	0.83	0.52	1.33
≥ 10 children	1.68**	1.17	2.42	1.25	0.77	2.02	0.67*	0.46	0.99	0.92	0.35	2.42
**Respondent’s education**												
No education (reference)												
Primary	1.19**	1.06	1.35	1.01	0.86	1.19	1.55***	1.38	1.75	2.04***	1.53	2.72
Secondary	1.45***	1.29	1.64	1.25**	1.08	1.46	1.65***	1.47	1.86	1.49***	1.11	2.01
Higher	2.01***	1.73	2.34	1.51***	1.26	1.81	2.23***	1.91	2.61	2.08***	1.48	2.91
**Husband’s education**												
No education (reference)												
Primary	0.94	0.41	3.24	0.89	0.76	1.04	1.07	0.94	1.21	1.02	0.70	1.47
Secondary	1.02*	0.37	3.21	1.10	0.98	1.24	1.14*	1.03	1.26	1.15	0.85	1.55
Higher	1.56*	0.55	4.70	1.65***	1.47	1.85	1.29***	1.14	1.46	1.43*	1.03	1.99
**Access to information**												
No (reference)												
Yes	1.44*	1.22	1.94	0.92	0.80	1.07	1.39***	1.26	1.54	1.57***	1.28	1.93

Furthermore, the results of the multivariable logistic regression model with dependent variable of “women empowerment” indicated that, after adjustment, almost all of the predictor variables were significantly associated with women’s empowerment. It was revealed that women’s empowerment increased if a woman was the head of household (AOR = 2.18, 95% CI: 1.89–2.53 in 2012–13; AOR = 2.46, 95% CI: 2.16–2.81 in 2017–18). Similarly, 2012–13 data indicated that women living in urban areas were 1.18 (95% CI: 1.08–1.29) times more likely to be empowered than those living in rural areas. The likelihood of women with children were more empowered than women with no children. The data indicated that women with 4–6 children were most likely to be empowered (AOR = 1.90, 95% CI: 1.63–2.22 in 2012–13; AOR = 1.17, 95% CI: 1.01–1.36 in 2017–18). The results highlighted a significant association between occupation and women’s empowerment, wherein women in both skilled and unskilled employment were more likely to be empowered than unemployed women.

Access to information was positively associated with women’s empowerment. The husband’s education and women’s empowerment did not appear to be significantly associated in the adjusted odds ratio model, although a husband with higher education was significantly associated in the binary logistic regression (Table 5).

**Table 5:** *Multivariable logistic regression of factors associated with women empowerment (n = 13,558 in PDHS 2012*–*13 and n = 15,068 in PDHS 2017*–*18)*

	PDHS 2012–13			PDHS 2017–18		
	AOR	95% CI		AOR	95% CI	
		Lower	Upper		Lower	Upper
**Age in years**						
15–19 (reference)						
20–24	1.33	0.69	2.56	1.31*	1.02	1.67
25–29	2.22**	1.16	4.23	1.77***	1.40	2.25
30–34	3.00***	1.54	5.86	2.45***	1.92	3.12
35–39	4.26***	2.15	8.43	2.82***	2.21	3.62
40–44	7.31***	3.52	15.18	3.55***	2.74	4.59
45–49	4.35***	2.11	8.96	4.88***	3.75	6.36
**Type of place of residence**						
Rural (reference)						
Urban	0.72*	0.54	0.95	0.94	0.86	1.03
**Sex of household head**						
Male (reference)						
Female	2.05**	1.28	3.28	2.46***	2.16	2.81
**Wealth index**						
Poorest (reference)						
Poorer	1.60***	1.20	2.13	1.19**	1.05	1.34
Middle	1.58**	1.14	2.19	1.27***	1.11	1.46
Richer	1.63*	1.10	2.42	1.31***	1.13	1.52
Richest	1.56*	0.94	2.60	1.33***	1.13	1.57
**Occupation of respondent**						
Unemployed (reference)						
Unskilled	1.97***	1.74	2.24	1.76***	1.39	2.22
Skilled	1.91***	1.66	2.19	1.43***	1.21	1.69
Managerial	2.09***	1.63	2.69	2.00***	1.71	2.34
**Number of children**						
No children (reference)						
1–3 children	2.20***	1.57	3.08	1.22**	1.07	1.39
4–6 children	2.68***	1.83	3.93	1.17**	1.01	1.36
7–9 children	1.60*	0.99	2.60	0.82*	0.67	1.00
≥ 10 children	1.36	0.53	3.48	0.76	0.52	1.11
**Respondent’s education**						
No education (reference)						
Primary	1.05	0.75	1.48	1.58***	1.40	1.78
Secondary	1.86**	1.20	2.87	1.66***	1.48	1.87
Higher	2.51***	1.55	4.05	2.33***	1.99	2.71
**Husband’s education**						
No education (reference)						
Primary	1.46	0.50	4.25	0.98	0.86	1.12
Secondary	1.42	0.49	4.13	1.02	0.92	1.14
Higher	1.89	0.65	5.50	1.10	0.97	1.25
**Access to information**						
No (reference)						
Yes	1.34*	1.02	1.77	1.25***	1.13	1.39

Note: All these variables were adjusted for region, income, and employment to perform multivariable logistic regression analysis to obtain adjusted odds ratios. AOR = Adjusted odds ratio, CI = Confidence interval (*p < 0.05; **p < 0.01; ***p < 0.001)

## Discussion

The results of this study reveal that almost all of the predictor variables are significantly associated with decision-making and most of these with ownership. Furthermore, results indicate that women’s empowerment is well predicted by demographic, economic, social, and information-exposure factors. It was noted that women having higher education, living in urban areas, and having access to information were more likely to be empowered. Likewise, women belonging to older age group, being the head of household, earning more than their husbands, involved in paid work, belonging to the rich class, and having children, were more likely to be empowered.

The results highlighted a significant association between a woman’s age and her empowerment, i.e. women’s empowerment increased with increasing age. These results are also supported by various other studies conducted in South Asia, including Nepal [30], Bangladesh [31], and India [32]. One of the reasons identified for this trend in age and empowerment is attributed to power relations within the household [33]. In the case of Pakistan, marriages are usually arranged at a young age – almost half of all women are married before the age of 20 years [34]. In this context, childbearing, particularly before the age of 18 years, is detrimental to mother and child, due not only to adverse reproductive health outcomes but also to social adjustments [35]. These women are mostly deprived of the opportunity to pursue other activities, such as schooling or employment [36].

Women’s place of residence was also significantly associated with empowerment. Similar to previous studies, the results highlighted that women living in urban areas were more empowered than their rural counterparts [37, 38]. Poverty-stricken rural women face a lack of economic opportunities and independence that pushes them another step away from decision-making [39].

The findings highlighted women’s education as a very strong predictor of empowerment. Since education enhances empowerment through increased skills, self-confidence, and knowledge [40, 41], and improves employment opportunities, as well as bringing income and healthcare-seeking mobility [42], highly educated women were found to be more empowered than those with low or no education. Arguably, housewifery is an expected gender role for women in Pakistan that diminishes educational opportunities for many young girls, particularly in rural areas [43, 44]. The study’s findings revealed that education of both spouses has a significant association with women’s empowerment [45]. By the same token, higher levels of education for both spouses result in more egalitarian decision-making within the household [46].

One of the most important results was the significant association between number of children and empowerment. Women with children, as compared to women without children, were more empowered, with the most highly empowered being those who had 4–6 children. The DHS data for Namibia and Zambia also highlight similar trends [47]. Similarly, DHS from Zimbabwe highlights a positive association between the number of male children and women’s empowerment [48]. Although the number of children, especially male ones, may solidify familial bonds and bring out a rather empowered guardian of her children aspect in a mother’s personality, it certainly cannot be taken as a policy outlook of empowerment in the same way as education, employment, and political participation.

Women’s empowerment increased consistently with increasing household wealth index. Similar results have also been reported from various other Southeast Asian countries, including Cambodia, Indonesia, the Philippines, and Timor-Leste [31]. In Pakistan, women stand low on the wealth index because their rights to inheritance and the ownership and management of property are poorly realized [28, 49]. Concomitantly, research indicates that women’s access to property and household resources does not guarantee empowerment; rather, it is control over those resources – ownership – that empowers women [50].

In the case of inheritance of property, Muslim countries, including Pakistan and Muslim-dominated areas of various other countries, enshrine the Islamic law of inheritance (Sharia) alongside the state laws [51]. Nonetheless, as in Pakistan, woman’s right to inheritance is poorly realized in the majority of the most populous Muslim countries/communities. This is mainly due to patriarchal customs and socio-cultural dynamics that give preference to men over women. Against the given backdrop, there is a dire need to introduce legal reforms, accompanied by viable administrative actions, across the Muslim countries, and particularly in Pakistan. Such an affirmative action could help to reduce gender-based discrimination and improve a range of socio-economic outcomes for women [52, 53].

Additionally, women’s productive employment is abysmally low, particularly in white-collar jobs and in rural areas [54]. Mostly, women are engaged in the informal economy, which usually does not allow them to play an equal role with men to add to their family’s wealth [55]. Moreover, women in the bottom strata of society struggle merely to cope with their sheer poverty and to manage their subsistence [56]. There is a strong need to enforce existing laws of ownership and inheritance and devise policies that encourage women’s employment.

According to the study results, women’s paid work had a positive and significant association with empowerment. Women involved in paid work were more likely to be empowered within the household than women with no paid work. The study’s findings also revealed that women working as skilled labourers and in managerial positions were the most empowered. These findings are supported by numerous studies, including DHS data from various Southeast Asian countries [31, 57]. The greater empowerment of skilled working women can be attributed to their greater freedom of movement and financial independence [58].

By contrast, women who undertake unpaid work as part of sharing or shouldering responsibilities are usually neither recognized by their family nor considered as a contribution to the household or state economy [59]. In this context, the “gender-disaggregated analysis of impact of the budget on time use” is one of the tools of “gender responsive budgeting” (GRB), which stipulates that time spent by women in so-called “unpaid work” is considered in budgetary policy analysis [60]. In this context, in a society like Pakistan, where the work done by women is mostly taken for granted and not accounted for, there is a need to adopt GRB in order to elevate women’s status.

Women residing in female-headed households were more likely to be empowered than their counterparts dwelling in male-headed households. A study conducted with rural Nigerian women showed similar results [61]. Likewise, another study using data from the Pakistan Integrated Household Survey established that women living in female-headed households were more empowered than those living in male-headed households, mostly owing to their greater participation in household decision-making [62]. A woman-headed household does not imply the absence of men or their support in the household. The literature indicates that the involvement of both men and women in household decision-making contributes to the improved wellbeing of both the household and society [63].

The findings of this study establish an association between women’s access to information and empowerment within the household. It was noted that women having access to various information sources, including radio, television, and newspapers, were more likely to be empowered than women with no access to information. Nonetheless, women’s access to information in Pakistan is typically very low compared to that of their male counterparts. In principle, women with more information can be better aware of household needs and contribute more positively to household decision-making for the welfare of their family, particularly children [22]. Hence, information is a potent ingredient in ensuring women’s greater awareness and participation in public affairs [64].

The limitation that applies to this study is due to its cross-sectional design, which does not allow for causal conclusions. However, temporality can be established between women’s empowerment and various factors examined here. A further limitation is that data was assessed by interviewers, where socially desirable answers given by the women could lead to bias. Future studies may involve collection of primary qualitative data on the issue to draw a comparative picture of the present study.

## Conclusions

This study provides useful insights into women’s empowerment and its various determinants within Pakistan. The results are drawn from a large, and hence generalisable, body of data, which consistently predicts a significant association between the studied demographic, economic, familial, and information-exposure factors, and women’s empowerment. The results of the present study suggest the importance of enforcing policies to restrict girl-child marriages, which adversely affect girls’ reproductive health and social well-being. The feminized poverty in Pakistan also needs to be alleviated through targeted action, particularly in rural areas where women’s access to information, employment, and inheritance is mostly denied. Women’s education and employment are the areas identified as requiring gender-based equal opportunities initiatives through a policy to enhance the socioeconomic status of women and achieve development at the national scale. Therefore, greater efforts are required to improve women’s access to employment and educational opportunities. There is also an urgent need to use mass communication and education campaigns to change community norms and values that discriminate against women. These campaigns must convey the potential contribution of women to the overall welfare of both their families and the wider society.

## Data Availability

The present study used raw data of the Pakistan Demographic and Health Survey 2012–13 and 2017–18. The data that support the findings of this study are freely available from Measure DHS to authors upon submission of request.

## Abbreviations

AOR: Adjusted odds ratio
CI: Confidence interval
DHS: Demographic and Health Survey
GRB: Gender Responsive Budgeting
ICT: Islamabad Capital Territory
OR: Odds ratio
PDHS: Pakistan Demographic and Health Survey
SPSS: Statistical Package for Social Sciences
TV: Television
